# The Work-ability Support Scale: Evaluation of Scoring Accuracy and Rater Reliability

**DOI:** 10.1007/s10926-013-9486-1

**Published:** 2013-12-12

**Authors:** Lynne Turner-Stokes, Joanna Fadyl, Hilary Rose, Heather Williams, Philip Schlüter, Kathryn McPherson

**Affiliations:** 1 Department of Palliative Care, Policy and Rehabilitation, School of Medicine, King’s College London, London, UK; 2 Regional Rehabilitation Unit, Northwick Park Hospital, Watford Road, Harrow, London, Middlesex HA1 3UJ UK; 3 Person Centred Research Centre, Health and Rehabilitation Research Institute, AUT University, Auckland, New Zealand; 4 School of Health Sciences, University of Canterbury, Christchurch, New Zealand; 5 School of Public Health and Psychosocial Studies, AUT University, Auckland, New Zealand; 6 School of Nursing and Midwifery, University of Queensland, Brisbane, Australia

**Keywords:** Rehabilitation, Vocational, Needs assessment, Reliability and validity

## Abstract

*Purpose* The Work-ability Support Scale (WSS) is a new tool designed to assess vocational ability and support needs following onset of acquired disability, to assist decision-making in vocational rehabilitation. In this article, we report an iterative process of development through evaluation of inter- and intra-rater reliability and scoring accuracy, using vignettes. The impact of different methodological approaches to analysis of reliability is highlighted. *Methods* Following preliminary evaluation using case-histories, six occupational therapists scored vignettes, first individually and then together in two teams. Scoring was repeated blind after 1 month. Scoring accuracy was tested against agreed ‘reference standard’ vignette scores using intraclass correlation coefficients (ICCs) for total scores and linear-weighted kappas (kw) for individual items. Item-by-item inter- and intra-rater reliability was evaluated for both individual and team scores, using two different statistical methods. *Results* ICCs for scoring accuracy ranged from 0.95 (95 % CI 0.78–0.98) to 0.96 (0.89–0.99) for Part A, and from 0.78 (95 % CI 0.67–0.85) to 0.84 (0.69–0.92) for Part B. Item by item analysis of scoring accuracy, inter- and intra-rater reliability all showed ‘substantial’ to ‘almost perfect’ agreement (kw ≥ 0.60) for all Part-A and 8/12 Part-B items, although multi-rater kappa (Fleiss) produced more conservative results (mK = 0.34–0.79). Team rating produced marginal improvements for Part-A but not Part-B. Four problematic contextual items were identified, leading to adjustment of the scoring manual. *Conclusion* This vignette-based study demonstrates generally acceptable levels of scoring accuracy and reliability for the WSS. Further testing in real-life situations is now warranted.

## Introduction

Broadly defined, vocational rehabilitation is “anything that helps someone with a health problem to stay at, return to or remain in work” [1]. Following illness or injury, vocational rehabilitation has an important role in assisting return to work for those who are able, and withdrawal from work for those who are unable to continue in their previous employment. An important requisite for both these tasks is the accurate assessment of work-ability.

Work-ability is a concept that can be broadly defined as “the match between the physical, mental, social, environmental, and organisational demands of a person’s work and his or her capacity to meet these demands” [2] (p 1173). Measurement of work-ability therefore requires consideration of a range of factors, including physical ability to perform tasks, ability to cope with the cognitive/communication demands of the job, and to function appropriately in the social and environmental context of the work.

Although a number of measurement tools have been developed for work-ability, our recent review of these highlighted a number of limitations which limit their use in clinical practice [2]. Despite the multifactorial nature of work-ability, the majority of measures focus predominately on ‘physical’ ability to do tasks. They rarely take into account the role of contextual factors or key stakeholders, and few tools have actually been developed with an intention to aid or assist in rehabilitation planning.

The Work-ability Support Scale (WSS) is a new measure that has been developed as part of a long-standing international collaboration between the United Kingdom (UK) and New Zealand (NZ). We set out to develop a tool which would not only cover all the key factors that contribute to work-ability, but would also provide a practical resource for clinicians to use for planning vocational rehabilitation/support in the course of routine practice. An overview of the conceptualisation, design and development is being presented for publication elsewhere. In brief, it included a mixed methods design incorporating:

- a conceptual review;
- qualitative work to inform the provisional structure of the tool, item definition, scoring framework, and the manual for training and score derivation; and
- quantitative evaluation of psychometric properties.

The conceptual review is published [2] and the qualitative work underpinning item generation and evaluation of utility and usability is described in more detail elsewhere. In this paper, we describe an evaluation of scoring accuracy, intra- and inter-rater reliability as part of (c).

## Setting and Design

Development of the WSS involved an iterative process of testing and refinement. To extend the eventual generalizability and utility of the tool, this was conducted in two different health cultures and services settings—a local community-based vocational rehabilitation setting in New Zealand and a tertiary post-acute, primary hospital-based rehabilitation service in the UK. Two rounds of evaluation were undertaken during that process.

- A preliminary round of inter-rater reliability testing undertaken in New Zealand (round 1) utilising case histories, led to the identification and adjustment of weaker items within the tool.
- Following revision based on the results of the preliminary round and the development of a set of test vignettes with reference standards, further evaluation was undertaken in the UK (round 2) as the penultimate stage in its production, to assess scoring accuracy as well as inter- and intra-rater reliability.

## The Work-ability Support Scale (WSS)

The WSS is a tool designed to:

- Assess the individual’s ability to work and support needs in the context of their normal work environment, following the onset of acquired disability,
- Support decision-making with regard to vocational rehabilitation.

It encompasses the complexity of physical, cognitive and behavioural challenges that are typically associated with neurological disability. However, it also has application in the more general context of work-related disability.

In its clinical application, the WSS is intended to be applied by a clinician on the basis of direct observation and interview with managers/co-workers in the course of a work-based vocational assessment. Alternatively, however, it may also be applied as part of screening to determine whether return to work is likely to be possible at an earlier stage in recovery. In this case, rating would be based on the anticipated performance in the workplace, deduced from off-site assessment of function in relation to a description of the individual’s work-based activities and job role. This type of application has been used to useful effect in a number of work-planning scenarios in the UK setting, including:

- where withdrawal from work was considered the only appropriate option, and a timely decision was required to avoid the individual losing out on pension payments (the WSS identifying that the likely level of work support required would be unsustainable).
- where the individual and/or their family had difficulty accepting that return to their current job role was not a realistic option. Scoring of the WSS supported dialogue between the patient and team as a step towards accepting exploration of alternative work and life roles.
- where return to work was considered feasible, but a strong case had to be put forward to support an application for funding for vocational rehabilitation.

The conceptual design of the WSS was based on a 7-level scoring framework similar to that of the Functional Independence Measure (FIM) [3]. The FIM is the most widely used outcome measure for rehabilitation across the world, and this framework was chosen because clinicians are broadly familiar with this concept.

The WSS is divided into two main parts.

Part A is a 16-item scale divided into three domains of work-related function:

- Physical function (five items)
- Thinking and communicating (five items)
- Social/behavioural function within the work-place (six items).

Each item is rated on a standardised ordinal 7-level scoring system ranging from 7 (completely independent) to 1 (totally unable), with the levels between reflecting an increasing requirement for help/support and the consequent decrease in work productivity.

For example:

- At level 7, the individual manages all of that aspect of his/her work without help. He/she performs independently without undue effort, and without the need for job modification. He/she requires no more equipment or strategies than would be considered normal for the role. Work productivity is fully maintained.
- At level 1, he/she effectively unable or require so much supervision/support that work productivity would be minimal.

Part B is 12-item scale of contextual factors, relating to personal and environmental/support factors which may influence return to work. These are also divided into three domains:

- Personal factors
- Environmental factors
- Barriers to return to work.

Originally scored on a five-point scale, the scaling was adjusted after the first round of inter-rater testing to a simpler 3-point scale indicating the overall effect of the contextual factor (positive effect = +1, neutral or unknown effect = 0, negative effect = −1).

The final tool structure and scoring levels are given in “Appendix”. It is recommended that the WSS is always applied using the scoring manual to ensure scoring accuracy, and this may be downloaded freely from our website http://www.csi.kcl.ac.uk/wss.html.

### Case Histories and Vignettes

In line with previous studies of this kind [4, 5] we used case histories and test vignettes to describe various levels of independence in work-related function as the test material for the purpose of evaluation. The production of generalisable data on inter-rater reliability presents a challenge for tools that measure functional ability, because of the practical difficulty of assembling a large number of raters around an individual patient to observe the same task in real-life settings. Proxy materials, such as case histories, videos or vignettes are therefore frequently used for preliminary testing [6].

- A case history is a short monologue describing a range of different attributes and characteristics relevant to the domains of interest, from which the rater extracts the relevant information to apply a rating for each item in the measurement tool.
- A set of vignettes provides more concrete and specific descriptions of function, focussing on one item at a time, and fixing the level of ability within that item [7].

When several raters use case histories to apply a measure independently, variations in scoring levels may reflect (a) the information they extracted to rate a given item and (b) their interpretation of that information to fit within the cut-points of the scale. When vignettes are used, the information is more standardised, so that variation is attributable to (b) alone. In theory therefore vignettes should produce less inter-rater variation. However, this depends to some extent on how they are written, and on how closely the vignette description mirrors the language of the measurement tool.

## Round 1: Preliminary Evaluation of Inter-rater Reliability

The first round of evaluation was undertaken in NZ in 2009/10, in the context of community-based services. The design of this phase was based around multiple raters scoring case histories, which were written up from clinical assessments.

### **Case Histories**

A set of 30 case histories were written up by Authors JF and KM from detailed notes taken during clinical work site assessments. The assessments were conducted by trained occupational therapists working in a vocational rehabilitation setting, and the clients of these assessments gave informed consent for notes from their assessment to be anonymised and written up as case histories for the research. Each case history was about 800–1,500 words and contained information about the individual, their clinical condition, their work role and a range of information about their working ability, relationship with colleagues and other specific information that would enable the rater to score each item. The case histories spanned a range of neurological and musculoskeletal problems of varying severity leading to a range of cognitive and/or physical disabilities. They also included a wide range of jobs and work roles in different contexts, including indoor and outdoor occupations.

### **Raters**

Five occupational therapists were recruited from community-based services providing vocational rehabilitation. The raters were all experienced work-place assessors who had not previously been involved with the cases described in the histories.

All raters were novice to using the WSS, so underwent a 4-h training session which used a similar format as standard FIM-training in New Zealand. It included an overview of the tool and scoring structure, orientation to the scoring manual, and practice cases which were worked through using the scoring manual and discussed in groups.

After the training, each rater scored the 30 case histories over a four-week period, again using the scoring manual. The cases were randomly numbered to avoid any systematic bias, and each assessor was presented with the cases in a different order to avoid any effects based on the order of rating.

Raters gave a score for each item for each case history and made comments where they felt scoring decisions were difficult to make or item descriptions were ambiguous. These comments were subsequently analysed to identify remaining ambiguities in item description and scoring instructions. Based on this feedback, revisions were made to the affected items and further inter-rater testing with four raters was conducted on the modified items only.

### **Statistical Handling**

Inter-rater agreement was tested using the multi-rater method described by Fleiss [8]. Kappa coefficients for multiple raters (mK) were calculated using the Statistical Analysis software (SAS^®^) macro MKappa [9].

### Results

Table 1 shows an item-by-item analysis of inter-rater agreement. The majority of Part A items showed moderate to substantial agreement. In response to feedback, ‘*communication*’ was divided into two items (written and verbal); significant changes were made to three other items, and there was also some re-grouping within the subscales. However, the contextual items showed only fair agreement. Discussion indicated that the items in the contextual factors domain were too broad and very difficult to score, so a substantive restructuring of that part of the scale was undertaken. Following these revisions, five modified work functioning items and seven new contextual factors items were re-tested for inter-rater agreement, with modest improvements demonstrated. However, agreement was still only moderate for the contextual items and further adjustments were made, expanding the number of items and simplifying the rating to just three scoring levels indicating positive, neutral or negative impact.

**Table 1:** Results of preliminary evaluation of inter-rater agreement (round 1: New Zealand): item-by-item analysis

Item	Initial analysis		Further testing on revised items only		
	Fleiss kappa (mK) (95 % CI)	Landis and Koch interpretation		Fleiss kappa (mK) (95 % CI)	Landis and Koch interpretation
Physical function					
Physical and motor	0.69 (0.65–0.73)	Substantial	Unchanged	Not re-tested	
Sensory and perceptual	0.64 (0.58–0.70)	Substantial	Unchanged	Not re-tested	
Mobility and access	0.61 (0.56–0.66)	Substantial	Unchanged	Not re-tested	
			*Community mobility*	*0.49* (*0.37–0.62*)	*Moderate*
Stamina and pacing	0.56 (0.51–0.61)	Moderate	Unchanged	Not re-tested	
Thinking and communicating					
Cognitive	0.64 (0.59–0.69)	Substantial	Unchanged	Not re-tested	
Planning and organising	0.37 (0.32–0.42)	Fair	*Planning and organising*	*0.45* (*0.33–0.56*)	*Moderate*
Problem solving	0.51 (0.46–0.56)	Moderate	Unchanged	Not re-tested	
Communication	0.60 (0.54–0.65)	Moderate	*Communication* (*verbal*)	*0.50* (*0.38–0.62*)	*Moderate*
			*Communication* (*written*)	*0.31* (*0.16–0.45*)	*Fair*
Social behavioural					
Work practices/etiquette	0.43 (0.38–0.48)	Moderate	*Self presentation*	*0.29* (*0.22–0.36*)	*Fair*
Maintaining safety	0.58 (0.53–0.63)	Moderate	Unchanged	Not re-tested	
Interpersonal (clients)	0.63 (0.58–0.68)	Substantial	Unchanged	Not re-tested	
Interpersonal (colleagues)	0.60 (0.55–0.65)	Moderate	Unchanged	Not re-tested	
Interpersonal (managers)	0.52 (0.46–0.57)	Moderate	Unchanged	Not re-tested	
Instruction and change	0.48 (0.43–0.53)	Moderate	Unchanged	Not re-tested	
Contextual factors					
Transport	0.25 (0.19–0.30)	Fair	*Personal support outside the workplace*	*0.54* (*0.42–0.76*)	*Moderate*
Supports outside the workplace	0.39 (0.33–0.45)	Fair	*Professional support outside the workplace*	*0.59* (*0.42–0.76*)	*Moderate*
Attitudes and feelings towards work	0.31 (0.25–0.37)	Fair	*Employer factors*	*0.44* (*0.28–0.60*)	*Moderate*
Competing demands	0.37 (0.31–0.42)	Fair	*Attitudes and feelings towards work*	*0.41* (*0.25–0.58*)	*Moderate*
Knowledge, beliefs and expectations	0.31 (0.26–0.36)	Fair	*Relationship with boss/supervisor*	*0.77* (*0.62–0.92*)	*Substantial*
			*Competing demands*	*0.52* (*0.35–0.68*)	*Moderate*
			*Financial and legal factors*	*0.64* (*0.48–0.80*)	*Substantial*

Items that were revised are shown in italics, together with the results of re-testing

## Round 2: Evaluation of Scoring Accuracy, Inter- and Intra-rater Agreement

This further round of evaluation was undertaken in the UK in January 2011, during the penultimate stages of development, once the structure of the tool had stabilised. Conducted with primarily hospital-based clinicians, it sought to address the following:

- scoring accuracy of individuals and teams against the set of reference standard scores,
- inter-rater reliability for both individual and team scores, and
- intra-rater reliability for individual and team scores rated on two occasions 1 month apart.

### Vignette Development

A possible weakness of the case histories such as those used in round 1 is that they contain a large amount of information requiring considerable concentration and retention on the part of the rater. Scoring differences may arise from the raters using different information from within the history to judge the level for a particular item. For round 2 in the UK we therefore used a more targeted vignette-based approach, analogous to the ‘case studies’ that are used for training and accreditation of the FIM in the US, Australia and the UK [10]. A preliminary description is given of each hypothetical case, followed by a brief description in 50–100 words of their work-related function under each of the item headings in the WSS. This enables the vignette writers to ensure that each item is tested across the range of scores.

A series of vignettes was drawn up by authors KM and LTS. In order to mimic the complexity of cases seen in clinical practice [4], they were designed to represent a range of difficulty for scorers—some led to a clearly evident score when referring to the manual, and others were less clear, requiring the rater to decide between one or more possible scoring levels. During development of the vignettes, the two authors first rated them independently, and then conferred to agree a ‘correct’ or reference standard rating to be assigned to each vignette.

When designing the study, consideration was given to rater burden among practising clinicians and the feasibility of rating large numbers of vignettes within an acceptable time allocation. The final set of 196 vignettes used for this evaluation related to 7 case studies—7 × 16 = 112 vignettes for Part A items and 7 × 12 = 84 for contextual items. The item scores were purposively chosen to provide good coverage of the range of possible scores for each item.

### Vignette Rating

Six clinicians took part in the study. No specific training was provided, but by this time the tool had been in routine clinical use within this unit for some years. To be included, participants were required:

- to have clinical experience in neurological rehabilitation focussed on work-related function,
- to have some experience with rating the WSS, and
- to be available for the two rating occasions 1 month apart.

The six raters were all affiliated to a large regional specialist neurorehabilitation unit spanning hospital and community outreach services in London, UK. All raters were occupational therapists, but were selected to represent a range of experience, both clinically and in use of the WSS, i.e. we included both senior and junior staff. They were organised into two teams, again representing a range of experience, in an attempt to mimic the pattern of scoring ability normal in clinical practice.

On the first occasion (test time 1), each clinician rated the vignettes individually, without conferring, but with reference to the scoring manual. As in round 1, the vignettes were presented to each of the raters in a different order. The following week, they met to score the vignettes as a team. This process was repeated 1 month later (test time 2), leaving sufficient time to limit recall bias. According to the manual, if there is disagreement between team members when rating as a team, the lower score is recorded (as is also the convention on rating the FIM).

### Data Handling and Analysis

The literature contains many different approaches to the testing of agreement between and within raters, and as yet no universal approach has emerged.

- The percentage of agreement between raters provides a simple descriptive analysis, but can be misleading as it does not take into account the extent of agreement that is simply due to chance.
- Cohen’s kappa was introduced to adjust for chance agreement [11], but un-weighted kappas do not account for the ‘degree of disagreement’, where disagreement of one category may be acceptable but wider disagreements may not.
- Weighted kappa coefficients were introduced in the late 1960s to provide partial credit for scaled disagreement [12] and are recommended by the Medical Outcomes Trust to evaluate agreement between raters for ordinal scales [13]. This is particularly relevant for long ordinal scales with more than five or six scoring levels per item.
- Cohen’s kappa coefficients, however, test agreement between a single pair of raters. Fleiss 1971 proposed a method for generalisation of the kappa statistic to the measurement of agreement among multiple raters [14], although this un-weighted method does not take any weighting of disagreements into account.
- An alternative approach used by some authors is to treat each combination of raters as a separate data pair [4, 5]. This means however, that each kappa coefficient represents multiple pairwise comparisons, thus effectively representing an average across the group. This is a potential statistical limitation especially if the data are unbalanced.

In this round, we evaluated scoring accuracy, intra-rater reliability, and inter-rater reliability.

The dataset comprised a total of 42 individual ratings (6 raters × 7 cases) and 14 team (2 team × 7 cases) ratings at each of the test times 1 and 2.

- Scoring accuracy was evaluated through rater agreement with the reference standard scores. Data were pooled from test times 1 and 2 to generate n = 84 individual paired ratings for each item, and n = 28 team ratings per item.
- Inter-rater reliability was evaluated at test time 1 only, testing agreement between all possible combinations of rater pairs for individuals and teams. For individual raters, 15 possible pairings generated 105 pairs (15 × 7). As there were only two teams, team ratings generated just N = 7 pairs.
- Intra-rater reliability was evaluated for both individual and team scores between paired ratings test times 1 and 2, giving n = 42 individual and 14 team ratings per item.

WSS total scores were compared using intra class correlation coefficients.

For item-by-item analysis we used a number of the approaches described above.

- For all comparisons, we report descriptive statistics in terms of percentage of absolute agreement. This provides the opportunity to compare individual and team ratings, even though, at n = 7 and n = 14 respectively, the numbers of team ratings were too small for statistical analysis of inter- and intra-rater reliability.
- We also report agreement ±1 level for Part A (which include 7 possible scoring levels)—but not for Part B (which includes only 3 levels).
- For scoring accuracy and intra-rater reliability we report weighted kappas.
- For inter-rater reliability we report both weighted kappas between all pair combinations and also Fleiss’s kappa for multiple raters.

Linear-weighted Cohen’s kappa statistics were computed using STATA version 12.1 (Stata Corp., 2012), and interpreted according to Landis and Koch’s classifications [15]. The 95 % CI for these weighted kappa statistics were calculated using bootstrapping, employing the method and macro given by Reichenheim [16].

As with round one, inter-rater agreement was tested using the multi-rater method described by Fleiss [8]. Kappa coefficients for multiple raters (mK) were calculated using the Statistical Analysis software (SAS^®^) macro MKappa [9].

### Results

#### Scoring Accuracy

Overall scoring accuracy was evaluated by ICCs comparing total subscale scores with reference standard ratings. ICCs for individual ratings for Part A and B were 0.95 (95 % CI 0.78–0.98) and 0.78 (90.67–0.85) respectively. The ICCs for team ratings were 0.96 (0.89–0.99) and 0.84 (0.69–0.92).

An item-by-item analysis of scoring accuracy in relation to reference standard scores is shown in Table 2 using linear-weighted kappa (kw).

**Table 2:** Agreement between reference standard scores and ratings by individuals and teams, using pooled data from time 1 and 2

	Individual raters (n = 84 paired ratings)				Teams (n = 28 paired ratings)			
	% Absolute agreement	% Agreement ±1	Cohen’s kappa (kw)	95 % CI for kw	% Absolute agreement	% Agreement ±1	Cohen’s kappa (kw)	95 % CI for kw
*Part A*								
1. Physical and motor	89	98	0.94	0.90–0.98	96	100	0.98	0.94–1.00
2. Sensory and perceptual	76	100	0.89	0.84–0.93	93	100	0.97	0.91–1.00
3. Mobility and access	68	98	0.84	0.78–0.90	79	100	0.91	0.82–0.97
4. Community mobility	70	75	0.78	0.69–0.86	75	86	0.79	0.63–0.92
5. Stamina and pacing	47	87	0.71	0.63–0.78	54	89	0.74	0.61–0.86
6. Cognitive	75	86	0.83	0.76–0.90	82	86	0.86	0.70–0.96
7. Planning and organising	75	89	0.81	0.71–0.90	86	93	0.91	0.78–0.98
8. Problem solving	67	83	0.76	0.68–0.84	75	89	0.80	0.69–0.91
9. Communication (verbal)	65	98	0.81	0.74–0.88	71	100	0.85	0.76–0.93
10. Communication (written)	76	93	0.82	0.74–0.89	79	96	0.86	0.72–0.95
11. Self presentation	64	81	0.74	0.65–0.83	71	75	0.76	0.57–0.90
12. Maintaining safety	48	93	0.71	0.60–0.79	53	93	0.68	0.46–0.83
13. Interpersonal (clients)	52	77	0.71	0.63–0.79	61	89	0.80	0.69–0.90
14. Interpersonal (colleagues)	43	91	0.71	0.63–0.77	43	96	0.74	0.64–0.83
15. Interpersonal (managers)	58	91	0.75	0.68–0.82	71	96	0.85	0.75–0.93
16. Instruction and change	79	87	0.83	0.74–0.91	86	96	0.92	0.82–0.99
*Part B*								
1. Desire to work	88	–	0.80	0.70–0.90	96	–	0.94	0.80–1.00
2. Confidence	83	–	0.81	0.70–0.90	89	–	0.89	0.76–1.00
3. Realistic expectations	83	–	0.81	0.72–0.90	86	–	0.85	0.71–0.96
4. Personal support	79	–	0.72	0.56–0.84	75	–	0.61	0.27–0.85
5. Peer support in work	81	–	0.74	0.61–0.85	86	–	0.80	0.59–0.95
6. Employer contact	76	–	0.60	0.42–0.75	64	–	0.52	0.21–0.76
7. Employer flexibility	85	–	0.58	0.43–0.75	82	–	0.61	0.36–0.85
8. Vocational support	63	–	0.53	0.37–0.66	64	–	0.58	0.36–0.79
9. Competing demands	93	–	0.91	0.81–0.96	89	–	0.88	0.71–1.00
10. Financial disincentives	67	–	0.61	0.48–0.73	64	–	0.57	0.36–0.79
11. Legal	57	–	0.34	0.18–0.51	75	–	0.63	0.37–0.86
12. Other factors	86	–	0.70	0.55–0.86	79	–	0.56	0.31–0.87

Agreement between the test ratings and reference standard scores, was either in the ‘Substantial’ (kw 0.71–0.78) or ‘almost perfect’ (0.81–94) range for all Part A items confirming a high level of scoring accuracy for this part of the scale. Three of the 12 contextual items (‘Employer contact’, ‘Employer flexibility’, and ‘Vocational support’) achieved only moderate scoring accuracy (kw 0.53–0.60), and ‘Legal issues’ showed only slight agreement (kw 0.34 95 % CI 0.18–0.51) with the reference scores.

When vignettes were rated by a team, the scoring accuracy was marginally higher, achieving a mean 73 % (SD 15) absolute accuracy, compared with 66 % (SD 13) for individual ratings in Part A. For Part B, the mean percentage accuracy of team and individual ratings were similar (78 % (SD 11) and 79 % (SD 11) respectively).

The mean differences between the rater scores and the reference standard scores for the 16 Part A items were compared using paired t tests taking *p* ≤ 0.003 as the threshold for significant to account for multiple tests. This showed that individual raters scored significantly higher than the reference standard on 9/16 items (other item differences being non-significant). When rating in groups, however, scores were significantly higher for only one item (all other item differences again non-significant). This suggests that (a) discussion assisted more accurate scoring (in relation to the reference scores) and (b) raters were following the manual instruction to record the lower score where group members disagreed.

#### Inter-rater Reliability

Inter-rater reliability is summarised in Table 3.

**Table 3:** Inter-rater agreement between of individual and team ratings (time 1 only)

	Individual raters (n = 105 paired ratings)				Multi-rater analysis 6 rater pairs		Team ratings (n = 7 paired ratings)	
	% Absolute agreement	% Agreement ±1	Cohen’s kappa (kw)	95 % CI for kw	Fleiss kappa	95 % CI	% Absolute agreement	% Agreement ±1
*Part A*								
1. Physical and motor	82	95	0.90	0.85–0.94	0.79	0.71–0.87	86	100
2. Sensory and perceptual	74	100	0.88	0.83–0.92	0.68	0.59–0.77	86	100
3. Mobility and access	53	90	0.73	0.65–0.80	0.45	0.37–0.53	57	100
4. Community mobility	73	95	0.81	0.70–0.90	0.66	0.57–0.75	86	100
5. Stamina and pacing	67	91	0.83	0.77–0.88	0.61	0.52–0.69	57	86
6. Cognitive	59	83	0.72	0.64–0.80	0.49	0.40–0.58	100	100
7. Planning and organising	66	86	0.70	0.59–0.81	0.60	0.52–0.68	71	86
8. Problem solving	57	77	0.70	0.63–0.78	0.49	0.41–0.57	71	86
9. Communication (verbal)	68	90	0.77	0.70–0.84	0.53	0.44–0.63	57	100
10. Communication (written)	64	88	0.74	0.65–0.81	0.58	0.50–0.67	71	100
11. Self presentation	71	86	0.78	0.70–0.87	0.63	0.53–0.73	57	71
12. Maintaining safety	57	93	0.74	0.65–0.82	0.43	0.33–0.53	71	86
13. Interpersonal (clients)	54	79	0.73	0.65–0.79	0.43	0.34–0.52	100	100
14. Interpersonal (colleagues)	50	83	0.72	0.64–0.78	0.37	0.28–0.47	71	100
15. Interpersonal (managers)	49	73	0.63	0.53–0.71	0.34	0.25–0.43	57	100
16. Instruction and change	58	78	0.69	0.59–0.78	0.47	0.37–0.56	100	100
*Part B*								
1. Desire to work	85	–	0.67	0.56–0.78	0.63	0.48–0.79	86	–
2. Confidence	73	–	0.63	0.51–0.76	0.56	0.42–0.71	86	–
3. Realistic expectations	81	–	0.76	0.65–0.85	0.67	0.51–0.83	100	–
4. Personal support	83	–	0.69	0.51–0.83	0.59	0.44–0.74	86	–
5. Peer support in work	76	–	0.68	0.55–0.79	0.62	0.47–0.76	100	–
6. Employer contact	63	–	0.22	0.00–0.39	0.17	0.02–0.31	43	–
7. Employer flexibility	80	–	0.50	0.34–0.64	0.49	0.33–0.65	86	–
8. Vocational support	57	–	0.41	0.27–0.56	0.34	0.21–0.48	57	–
9. Competing demands	92	–	0.91	0.84–0.96	0.86	0.70–1.00	71	–
10. Financial disincentives	79	–	0.73	0.61–0.82	0.63	0.48–0.79	42	–
11. Legal	43	–	0.11	0.00–0.27	0.07	0.00–0.22	57	–
12. Other factors	84	–	0.69	0.54–0.82	0.71	0.57–0.85	57	–

ICCs for individual inter-rater agreement at test time 1 were 0.97 (95 % CI 0.96–0.99) for Part A and 0.72 (0.50–0.93) for Part B.

Using linear-weighted kappas, agreement between individual raters ranged from kw 0.63–0.90 for all Part A items confirming a high level of inter-rater reliability. In Part B, ‘Employer contact’ and ‘Vocational support’ again achieved only moderate levels of agreement (0.41 and 0.22 respectively) and ‘Legal issues’ showed very poor agreement (kw 0.11 (95 % CI −0.05 to 0.27)).

Inter-rater reliability was marginally higher for team ratings mean 73 % (SD 15) absolute agreement compared with 63 % (SD 9) for individual ratings in Part A. For Part B, percentage accuracy of team and individual ratings were similar [76 (SD 21) and 75 (SD 14)]. Kappa coefficients were not calculated because of the small number of paired team ratings.

In Table 3 we have also included an analysis of Fleiss multi-rater kappas. These (unweighted) kappa coefficients are significantly lower than the linear-weighted Cohen’s kappa statistics for the same dataset—mK for Part A ranged from 0.07 to 0.79, and for Part B from 0.07 to 0.86. They are included to highlight this difference (see “Discussion” section). Although not strictly comparable with the round 1 analysis (because of the different number of cases in the two evaluations) they give a broad indication that agreement is similar to that seen in round 1 in the physical and cognitive domains, but somewhat lower in the social/behavioural domain.

Despite the lower values compared with linear weighted kappas, the mK coefficients generally reflect a similar pattern, identifying the same three poorly performing contextual items—Employer contact, Vocational support and Legal issues.

#### Intra-rater Reliability

Intra-rater reliability is summarised in Table 4.

**Table 4:** Intra-rater agreement between of individual and team ratings

	Individual raters (n = 42 paired ratings)				Team ratings (n = 14)	
	% Absolute agreement	% Agreement ±1	Cohen’s kappa (kw)	95 % CI for kw	% Absolute agreement	% Agreement ±1
*Part A*						
1. Physical and motor	91	95	0.91	0.82–0.96	93	100
2. Sensory and perceptual	71	100	0.87	0.79–0.93	86	100
3. Mobility and access	60	95	0.79	0.67–0.88	71	100
4. Community mobility	86	100	0.95	0.91–0.98	93	100
5. Stamina and pacing	71	97	0.87	0.79–0.93	79	93
6. Cognitive	76	90	0.85	0.74–0.94	93	100
7. Planning and organising	60	81	0.69	0.52–0.81	71	86
8. Problem solving	67	83	0.78	0.66–0.87	71	86
9. Communication (verbal)	81	97	0.89	0.80–0.96	86	100
10. Communication (written)	62	83	0.71	0.54–0.83	86	93
11. Self presentation	67	83	0.78	0.62–0.90	86	93
12. Maintaining safety	67	95	0.81	0.66–0.90	79	79
13. Interpersonal (clients)	62	74	0.73	0.62–0.86	79	86
14. Interpersonal (colleagues)	52	83	0.73	0.61–0.83	79	86
15. Interpersonal (managers)	57	88	0.73	0.60–0.83	79	86
16. Instruction and change	69	90	0.80	0.65–0.91	79	93
*Part B*						
1. Desire to work	86	–	0.74	0.63–0.91	93	–
2. Confidence	79	–	0.71	0.50–0.87	79	–
3. Realistic expectations	91	–	0.88	0.74–0.98	100	–
4. Personal support	91	–	0.82	0.58–0.97	79	–
5. Peer support in work	81	–	0.75	0.56–0.89	100	–
6. Employer contact	83	–	0.71	0.47–0.90	79	–
7. Employer flexibility	93	–	0.82	0.67–0.94	86	–
8. Vocational support	62	–	0.50	0.28–0.70	57	–
9. Competing demands	95	–	0.92	0.76–1.00	93	–
10. Financial disincentives	91	–	0.85	0.69–0.98	79	–
11. Legal	69	–	0.54	0.34–0.74	64	–
12. Other factors	79	–	0.54	0.30–0.81	79	–

ICCs for individual intra-rater agreement between test time 1 and 2 were 0.97 (95 % CI 0.95–0.99) for Part A and 0.89 (0.81–0.94) for Part B.

Item-by-item again analysis again showed ‘substantial’ to ‘almost perfect’ intra-rater agreement across all Part A items (kw 0.71–0.95) and were generally also satisfactory for Part B with the exception of two items—‘Vocational support’ and ‘Legal issues’ which showed moderate agreement (kw 0.50 and 0.54 respectively).

Intra-rater reliability improved for Part A items when clinicians rated in teams—mean 82 % (SD 8) agreement for team scoring compared with 69 % (SD 11) for individual rating. But once again, team and individual rating were similar for Part B items—mean 83 % (SD 10) and 82 % (SD 13) respectively.

## Discussion

In this article we have described an iterative process of evaluation and adjustment of the WSS, across two continents and in service settings spanning hospital and community. This approach was deliberately utilised to ensure that the final tool would have applicability across a range of health culture and experience.

The initial evaluation, based on case histories in the context of community-based rehabilitation in New Zealand, led to a significant restructuring and re-design to make the tool more useable by clinicians. The subsequent vignette-based study, centred on a primarily hospital-based service in the UK, demonstrated acceptable levels of scoring accuracy and reliability for the WSS Part A, both between raters and over time. Team ratings are expected to be more reliable, which may reflect both a learning effect and the instruction in the manual to record the lower score in the event of disagreement. In this study the tendency for teams to record lower item scores than individual raters suggests that they were following this instruction, which tends to reduce variation. Nevertheless, although team ratings were marginally more reliable than individual ratings, the latter still achieved very acceptable overall levels of accuracy and reliability and may therefore be considered adequate for clinical practice.

Part B (contextual items) proved more problematic to rate, despite the adjustments made after the preliminary round of evaluation. Rating of ‘Personal’ factors—such as having the desire or confidence to work, realistic expectations and personal support from family/friends achieved acceptable scoring accuracy and reliability in all cases. However ‘Environmental factors’—in particular ‘Employer contact and flexibility’ or ‘Vocational rehabilitation/support’ appeared to be more open to interpretation. ‘Barriers to return to work’ caused confusion because of the negative scoring system. However, even after this was corrected, the item concerning ‘Legal issues’ continued to show poor reliability. These scoring difficulties could either be due to the item descriptions or to the fact the vignettes for these items were harder to rate.

Vignettes were designed to mimic the complexity of cases seen in clinical practice with some being harder than others to rate, so the authors reviewed all the reference standard scores and discussed the ratings with the teams. These reflections identified some particular problems with the ‘zero’ and ‘positive’ scores for contextual items. For example, an on-going legal compensation claim was generally accepted as a negative influence on return to work, but the absence of such a case was variably interpreted as either ‘neutral’ or ‘positive’. Further adjustments have since been made to the scoring manual to instruct the rater to default to ‘zero’ scores for the contextual items, and only rate scores on either side of this if a given factor presents a clear positive or negative influence. Nevertheless, the findings presented here across several rounds of evaluation suggest that the contextual items are (and probably always will be) vulnerable to variable interpretation. Whilst clinicians agree that these are important factors to take account of in individual care planning, for the purpose of measurement, we suggest they should be used as a clinical checklist alongside the WSS, rather than as an integral part of the measurement tool.

In this study, we also explored a variety of approaches to measuring agreement. Because the WSS items comprise seven scoring levels, weighted kappas were considered to be most relevant and we also reported percentage of agreement ±1 scoring level. As may be expected, the weighted kappa statistics provided an estimate of agreement somewhere between the percentage of ‘absolute agreement’ and ‘agreement ±1’. For inter-rater reliability, there was some concern that multiple pairwise comparisons using linear weighted kappas may give spuriously high results, and for this reason we also applied the (un-weighted) Fleiss multi-rater method which gave substantially more conservative estimates of agreement—again as would be expected. The future design of a weighted method for calculating multi-rater kappa statistics would be a welcome statistical development, but to our knowledge no such technique currently exists. In the meantime, these differences highlight the importance of reporting the statistical methods used, as they may otherwise lead to significantly different conclusions about the reliability of tool performance.

Our findings must be interpreted in the light of some clear limitations to the study.

1. Vignettes were chosen in this evaluation to provide a stable, fictional presentation of a patient’s functional ability. This ensures that different raters are basing their scores on the same information. They do, however, provide a limited insight into the patient’s holistic ability, and cannot entirely replace field testing.
2. The vignette sample size was strictly suboptimal. The computation of Cohen’s kappa values is often said to require a sample size of 2K^2^ [4] which in this context would be 98. There is always a balance to be found between the use of hard-pressed clinicians’ time, and obtaining optimal numbers for statistical analysis. Introducing more vignettes would have reduced the number of volunteers, so compromise was therefore accepted. However, as increasing the sample size tends to increase the estimates of agreement, which were high even with a small sample, it is unlikely an expanded dataset would have altered our conclusions significantly.
3. The weighted kappa coefficients for inter- and intra-rater agreement were calculated on pooled samples for all six raters and thus effectively represent the average across the group. This is a potential statistical limitation if the data are unbalanced. At a clinical level, however, the pooling of data supports generalisability as the full range of inter-rater variability is represented.
4. The unit where the round 2 (UK) evaluation was undertaken was one of the locations in which the WSS was developed. Although the raters were purposively chosen to represent a range of experience, they would undoubtedly have had more experience with the WSS, than the average clinical centre using the scale, at least when it is first introduced.

Not withstanding these limitations, the findings provide preliminary evidence of reliability, which supports use of the WSS as a reproducible tool for assessing work-ability. Further testing in a wider sample and in the context of clinical application is now recommended.

## Appendix: The Work-ability Support Scale (WSS)

**Table:** Part A: 16 items—each item is rated on 7-level scoring system

*Physical*	
1. Physical and motor	Physical and motor skills required to do the job (e.g. lifting, dexterity, coordination, balance)
2. Sensory and perceptual	Sensory and perceptual skills required to do the job. Includes both sensory (e.g. vision) and perceptual (e.g. perception of differences between objects)
3. Mobility and access	Ability to move around in the work environment and access areas, facilities and equipment for the job
4. Community mobility	Moving around the community for work requirements, travelling to and from work and community mobility
5. Stamina and pacing	Ability to manage fatigue, and stamina to work through a normal working day
*Thinking and communicating*	
6. Cognitive	Ability to manage memory, attention, concentration, etc. requirements of the job
7. Planning and organising	Ability to initiate, plan and organise as required for the job
8. Problem solving	Ability to deal with non-routine or unexpected events in the workplace such as interruptions, problem solve and work to own initiative when things change
9. Communication (verbal)	Verbal communication ability including production and understanding of verbal communications
10. Communication (written)	Reading, writing and understanding of written material as required for the job
*Social/behavioural*	
11. Self presentation	Time keeping, appropriate dress and self presentation for the particular job role
12. Maintaining safety	Ability to maintain safety of themselves and others in the work environment
13. Interpersonal (clients)	Interpersonal skills, professional and social interaction with clients/customers
14. Interpersonal (colleagues)	Interpersonal skills, professional and social interaction with work colleagues
15. Interpersonal (managers)	Interpersonal skills, professional interaction with management
16. Instruction and change	Appropriate reaction to supervisory instruction and/or correction regarding work activities. Ability to correct errors, accept changes in work tasks, etc

**Table:** Scoring levels: Part A

*Independent*	
Level 7	Independence without modification No problem at any level with managing the requirements of the job
Level 6	Independence with modification Some consideration for time or effort* Or requires adaptation/strategies/equipment above the ordinary provided for the job in order to function independently Able to self-prompt/correct or to structure his/her own environment Minimal reduction in work productivity
*Supported working*	
Level 5	Supervision/set-up Requires someone else to set-up equipment or prompt on strategies Or externally structured work environment. Monitoring—with only occasional prompting/correction
Level 4	Minimal support Able to manage >75 % of the time in that aspect of the job Regular planned intervention or support only Work productivity only mildly affected
Level 3	Moderate support Able to manage more than half the time in that aspect of the job Infrequent** unplanned intervention on top of regular monitoring Work productivity moderately affected
Level 2	Maximal support Able to manage less than half the time in that aspect of the job Frequent unplanned intervention on top of regular monitoring Work productivity severely affected
Level 1	Constant support—or effectively unable Effectively unable or manages <25 % of the time Unplanned intervention many times a day
Unable to score	Unable to score due to insufficient information. More information required

* ‘Safety’ is not included in level 6 as maintaining safety is included as an item on its own merit

** Frequency of unplanned interventions not rigidly defined in terms of time—it may vary for different items

**Table:** Part B Contextual Factors

Item	Contents
*Personal factors*	
1. Desire to work	Does N want to return to/remain in work?
2. Confidence	Does N feel confident in their ability to cope with work?
3. Realistic expectations	Does N have realistic expectations with respect to his/her working ability and return to work?
4. Personal support	Is there support from friends and family for N to return to work?
*Environmental factors* (*within the work place*)	
5. Peer support in work	Is there support from N’s workmates colleagues for N to return to work?
6. Employer contact	Is there contact with N’s employers with respect to return to work?
7. Employer flexibility	Is the employer willing to take positive steps to facilitate N’s return to work? (e.g. making adaptations to the job, the workplace etc.)
8. Vocational support/rehabilitation	Is there formal support from external services to coordinate return to work? (e.g. vocational rehab, disability employment service, case manager etc.)
*Barriers to return to work* (*Note negative scoring for this section*—*use score sheet*)	
9. Competing demands	Are there issues outside of work that potentially conflict with work commitment?
10. Financial disincentives	Are there any financial barriers to return to work? (e.g. insurance/unemployment benefits)
11. Legal	Are there any legal issues which present a barrier to N returning to work? (e.g. ongoing litigation)
12. Other factors	Are there any other factors positive or negative affecting N’s ability to return to/remain in work?

**Table:** Scoring levels: Part A

Scoring	Description	Not scored
+1	Positive effect	
0	Neutral/not sure/not applicable	More information needed
−1	Negative effect	
